# ^1^H, ^15^N backbone assignment and comparative analysis of the wild type and G12C, G12D, G12V mutants of K-Ras bound to GDP at physiological pH

**DOI:** 10.1007/s12104-019-09909-7

**Published:** 2019-08-29

**Authors:** Gyula Pálfy, István Vida, András Perczel

**Affiliations:** 1 Laboratory of Structural Chemistry and Biology, Institute of Chemistry, Eötvös Loránd University, 1/a. Pázmány Péter stny, Budapest, H-1117 Hungary; 2 MTA-ELTE Protein Modeling Research Group, Institute of Chemistry, Eötvös Loránd University, 1/a. Pázmány Péter stny, Budapest, H-1117 Hungary

**Keywords:** Ras, G12C, G12D, G12V mutants, Cancer, NMR, Combined chemical shifts analysis

## Abstract

K-Ras protein is a membrane-bound small GTPase acting as a molecular switch. It plays a key role in many signal transduction pathways regulating cell proliferation, differentiation, survival, etc. It alternates between its GTP-bound active and the GDP-bound inactive conformers regulated by guanine nucleotide exchange factors and GTPase activating proteins. Its most frequent oncogenic mutants are G12C, G12D, and G12V that have impaired GTPase activity, thus induce malignant tumors. Here we report the resonance assignment of the backbone ^1^H and ^15^N nuclei of K-Ras wildtype, G12C, G12D and G12V proteins’ catalytic G domain (1–169 residues) in GDP-bound state, and ^13^C of backbone and side chains of G12C mutant at physiological pH 7.4. Triple resonance data were used to get secondary structure information and backbone dynamics of G12C, the best-known drug target among K-Ras mutants. Simultaneous investigation of G12C, G12D and G12V mutants, along with the wild type form at the very same conditions allowed us to perform a comprehensive analysis based on the combined chemical shifts to reveal the effect of mutation at G12 position on structure. Intriguingly, the G12C and G12V mutants found to be structurally very similar at the three most important regions of K-Ras (P-loop, Switch-I, Switch-II), while the G12D mutant significantly differs at P-loop and Switch-II from the wildtype as well as G12C and G12V mutants. However, in Switch-I it hardly deviates from the wildtype protein.

## Biological context

Kirsten Ras (K-Ras), named after Werner H. Kristen (Kirsten and Mayer 1967; DeFeo et al. 1981), is one of the most frequent oncoproteins in human cancer. It belongs to the Ras subfamily of small GTPases and acts like a molecular switch regulating molecular pathways associated with cell growth and proliferation (Karnoub and Weinberg 2008; Ostrem and Shokat 2016). Beside other Ras proteins, like H-Ras and N-Ras, the Ras subfamily includes the two alternative splice product of KRAS gene, K-Ras4A and K-Ras4B. Ras proteins are highly homologous, membrane-localized GTPases, which contain guanosine nucleotide-binding domain (G domain of ~ 20 kDa) at the *N*-terminus and a short hypervariable membrane targeting region at the *C*-terminus, called a CAAX box. The G domain consists of two lobes, the effector (M1–N86 residues) and the allosteric lobe (T87–H166 residues). Nonetheless, distinct regions of the effector lobe are engaged in GTP hydrolysis and nucleotide exchange, like the P-loop (G10–S17 residues), Switch-I (Q25–Y40 residues) and Switch-II (D57–G75 residues) (Boriack-Sjodin et al. 1998). As a molecular switch, Ras proteins alternate between GDP-bound inactive and GTP-bound active conformations (Vetter and Wittinghofer 2001). As a consequence of its activation, Ras triggers an avalanche of serine/threonine kinases which leads to cell growth and proliferation via downstream signaling and transcriptional activation. Albeit Ras proteins carry both intrinsic GTPase and GDP/GTP exchange activity, the Ras cycle is controlled by guanine nucleotide exchange factors (GEFs) and GTPase-activating proteins (GAPs). GEFs promote the exchange of the nucleotide substrate, GDP to GTP, causing the activation of Ras. GAPs rapidly inactivate the protein by the acceleration of GTP hydrolysis.

In most of the cases, Ras mutations cause inhibition of inactivation by GAPs and decrease intrinsic GTP hydrolysis. Amongst the three most frequent Ras family member, K-Ras is the most commonly mutated, its mutations occur in 20% of all human cancers. 80% of all K-Ras mutations occur at the G12 position (Prior et al. 2012). The three major K-Ras mutations in this position are found to be the G12C, G12D and G12V (the occurrence is 16%, 40% and 26% of all G12 mutations), and their GTP autohydrolysis rates are reduced compared to the wildtype (wt) form in the order of listing (Hunter et al. 2015). Although K-Ras was considered to be undruggable for 30 years, it came into the spotlight again, when novel chemoselective and mutant specific inhibitors for the G12C mutant such as ARS-853 and ARS-1620 were discovered (Ostrem et al. 2013; Janes et al. 2018). Here we present the comparison of the assignment for the ^1^H, ^15^N-HSQC spectra of the three major K-Ras mutants together with the wt in their GDP-bound state under the very same conditions.

### NMR spectroscopy

Ras proteins were investigated by NMR spectroscopy in a number of studies, while previously mostly the H-Ras, nowadays K-Ras4B and variants were assigned, with different nucleotides and sample conditions. In the earlier studies, assignment of H-Ras(1–171) was done in GDP- and GTP-analogue GppNHp-bound form at acidic pH (~ 5.5) at various temperatures between 293 and 318 K (Muto et al. 1993; Ito et al. 1997). Based on these results ^1^H, ^15^N-HSQC assignments were performed for the same constructs at near physiological pH 7.5 and 298 K (Smith et al. 2013). The full assignment deposited into Biological Magnetic Resonance Data Bank (BMRB) including backbone and side-chain ^1^H, ^15^N and ^13^C chemical shifts of a slightly shorter construct, the wildtype H-Ras(1–166) were performed bound to both GDP (O’Connor and Kovrigin 2012, BMRB code: 18479) and GTP analogue GppNHp (O’Connor and Kovrigin 2012, BMRB code: 17678) at near physiological conditions: pH 7.2 at 293 K. An effector (cRaf1) bound form of this construct H-Ras(1–166) with GDP was assigned at pH 6.8 at 298 K (Araki et al. 2011, BMRB: 18461). Furthermore two mutations of H-Ras were studied, oncogenic mutant GDP-bound H-Ras-G12V(1–166) (Amin et al. 2016, BMRB code: 25730) at pH 7.5 (300 K), and a dynamically restricted version of GppNHP-bound Ras, H-Ras-T35S(1–166) at pH 6.8 (298 K) (Araki et al. 2011, BMRB code: 17610). The latter was investigated in a small molecule-bound form too (Shima et al. 2013, BMRB code: 18629) at pH 6.8 (278 K). Finally, partial ^13^C-assignment was done for the H-Ras-T35S-GppNHp bound to a small molecule (Matsumoto et al. 2018, BMRB: 36166).

Similarly to H-Ras, K-Ras4B was also studied extensively. Triple resonance assignment of the wildtype form of K-Ras4B(1–166) was completed in its GDP-bound form at physiological pH 7.4 (298 K) (Vo et al. 2013, BMRB code: 18529). Its near full-length versions were also studied, but at different pHs: GDP-bound K-Ras4B(1–188) at pH 6.5 (298 K) (Abraham et al. 2009, BMRB: 26635, HSQC spectrum assigned only) and the GDP-bound K-Ras4B(1–180) at pH 8.0 (296 K) (Gossert et al. 2011). *C*-terminal truncated versions of K-Ras4B(1–171) bound to the GTP-analogue GppNHp were assigned at acidic pH 5.9 (298 K) (Buhrman et al. 2011, BMRB code: 17785). A single mutant of this protein, the oncogenic mutant K-Ras4B-G12C(1–169) was assigned recently at near physiological pH 7.0 (298 K) in both GDP- (Sharma et al. 2018, BMRB: 27387) and GppNHp-bound form (Sharma et al. 2019, BMRB: 27472). Beyond these studies, several partially assigned ^13^C side chains either fixed in a nanodisc or other small molecule-bound form of K-Ras4B-GDP and K-Ras4B–GppNHp were described (Mazhab-Jafari et al. 2015; Fang et al. 2018, BMRB codes: 25114, 25115, 25116, 30400, 30401, 30403). No comprehensive analysis exists up to now on the wildtype K-Ras4B with its most frequent oncogenic mutant, such as G12C, G12D, and G12V by applying the very same experimental conditions to reveal the mutation induced structural effects.

## Methods and experiments

### Protein expression and purification

pET-15b plasmid, encoding K-Ras4B(1–169) wt, G12C, G12D and G12V fused to *N*-terminal His6-tag and tobacco etch virus (TEV) protease cleavage site, was transformed into the *E.coli* strain BL21(DE3). Transformed cells were grown in 2YT medium supplied with 0.1 g L^−1^ ampicillin with shaking at 180 rpm, 37 °C until an OD_600_ of 0.7–0.8 was reached. Cells were centrifuged, washed and resuspended in minimal medium containing 1 g L^−1^^15^NH_4_Cl and 4 g L^−1^ glucose (or 2 g L^−1^^13^C-glucose in the case of ^13^C/^15^N-labeling). Before induction, cells were shaken for 1 h and then protein expression was initiated by the addition of IPTG (isopropyl-β-d-1-thiogalactopyranoside) to a final concentration of 0.5 mM. After 3 h incubation time at 37 °C, cells were harvested by centrifugation, resuspended in lysis buffer (500 mM NaCl, 20 mM imidazole, 20 mM Tris pH 8.0) and stored at − 20 °C: cells were lysed by sonication afterwards. Unlysed cells and cell debris were removed by centrifugation. The target protein, solubilized in the supernatant was purified with a *three*-*step* procedure. *First*, the His-tagged fusion protein was separated from the other endogenous proteins by using nickel-nitrilotriacetic acid (5 mL; Ni–NTA) metal affinity chromatography column, which had been equilibrated with lysis buffer. The bound fusion protein was eluted with the same buffer containing imidazole (250 mM). The pools were dialyzed overnight against the dialysis buffer (500 mM NaCl, 20 mM imidazole, 1 mM DTT, 0.5 mM EDTA, 20 mM Tris pH 8.0). During the dialysis, the fusion protein was digested with His-tagged TEV. Purification results and digestion efficacy were monitored by sodium dodecyl sulfate–polyacrylamide gel electrophoresis (SDS-PAGE). *Second*, the TEV cleaved protein was loaded onto an affinity chromatography column again to separate the His-tag and TEV from the target protein. *Third*, the protein of interest was further purified by size-exclusion chromatography using Superdex 75 Increase column (10/300 GL) with PBS buffer (137 mM NaCl, 2.7 mM KCl, 10 mM Na_2_HPO_4_, 2 mM KH_2_PO_4_, pH 7.4) containing 10 mM EDTA. The freshly prepared and purified protein was then concentrated to 0.5–1 mM, flash-frozen in liquid nitrogen and stored at − 80 °C.

### NMR spectroscopy

The NMR samples of ^13^C, ^15^N-labeled (K-Ras4B-G12C(1–169)) or ^15^N-labeled proteins (K-Ras4B-wt(1–169), K-Ras4B-G12D(1–169) or K-Ras4B-G12V(1–169)) of 0.4–0.8 mM concentration contained 5 mM GDP, 10 mM EDTA, 15 mM MgCl_2_ (in 5 mM excess with refer to EDTA), 3 mM NaN_3_ in PBS buffer, 10% D_2_O with 1% DSS standard were added and pH was set to 7.4. NMR measurements were performed at 298 K on a Bruker Avance III 700 MHz spectrometer equipped with a 5 mm Prodigy TCI H&F–C/N-D, z-gradient probehead operating at 700.05 MHz for ^1^H, 70.94 MHz for ^15^N and 176.03 MHz for ^13^C nuclei. Temperature was calibrated by standard methanol solution (Ammann et al. 1982). All chemical shifts were referenced with respect to the internal ^1^H-resonance of DSS, while ^13^C, ^15^N-chemical shifts were referenced indirectly, by using the corresponding gyromagnetic ratios according to IUPAC convention (Wishart et al. 1995). Sequence-specific assignment of H^N^, N, C’, C^α^, C^β^ and H^α^ of the GDP-bound K-Ras4B-G12C(1–169) was done on the basis of ^1^H, ^15^N–HSQC, BEST-HNCA, BEST-HN(CO)CA, BEST-HNCACB, BEST-HNCO and (H)CC(CO)NH, HSQC-TOCSY, HSQC-NOESY spectra (Sattler et al. 1999; Schanda et al. 2006; Lescop et al. 2007). In case of the wt, G12D and G12V mutants ^1^H, ^15^N-HSQC and HSQC-NOESY spectra were collected and thus, the assignment obtained for G12C was transferred to obtain chemical shifts of backbone ^1^H and ^15^N. All spectra were processed with the Bruker TOPSPIN software and analyzed by using CARA (ETH Zürich) (Keller 2004).

## Assignments, data deposition and predictions

**Fig. 1:**
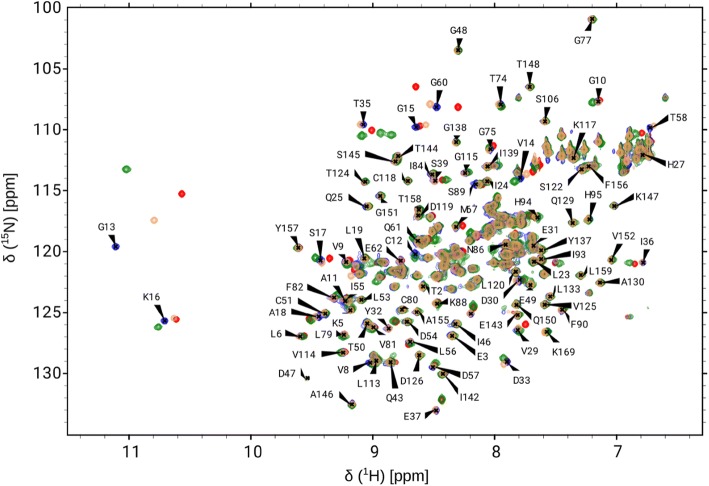
Overlaid ^1^H,^15^N-HSQC spectra of the GDP-bound form of K-Ras4B-wt (red), K-Ras4B-G12C (blue), K-Ras4B-G12D (green) and K-Ras4B-G12V (yellow) proteins. Labels refer to signals of G12C (only a partial assignment is indicated due to the crowded peaks in the center region of the spectra, the full assignment can be found in BMRB with accession number 27646)

All of the non-proline residues were assigned except T87 in case of wt, G12C and G12V mutants (97% of all residues). Assignment contains 96% of all G12D mutant residues because both M1 and T87 could not be assigned of the non-proline residues in this case (Fig. 1). The current assignment of K-Ras4B-wt-GDP agrees well with that of Vo and coworkers (Vo et al. 2013) completed at the same conditions (pH 7.4 and 298 K) although they have measured a TROSY version instead of the conventional HSQC experiment. The K167-E168-K169 tripeptide unit of the *C*-terminus was not included in their construct, furthermore both G48 and G77 appear as aliased peaks in their spectra, while we have determined the true chemical shifts of these resonances as well. The assignment of our G12C mutant agrees well with that of a recent work published by Sharma et al. (Sharma et al. 2018), even regarding ^13^C resonances. While their spectra were recorded at pH 7.0, ours at physiological pH 7.4. This slight difference could be the reason why T87 was not detected in our experiment due to line broadening. Therefore, N-H chemical shift of T87 is indeed pH sensitive. Furthermore, similar to Vo et al. G77 appears as an aliased peak in their work as well, while we have measured the true chemical shift of this residue within the mutant protein. The assigned chemical shifts have been deposited to the BMRB database (http://www.bmrb.wisc.edu) under the following entries: 27646 (G12C mutant), 27720 (wt), 27719 (G12D mutant) and 27718 (G12V mutant).

**Fig. 2:**
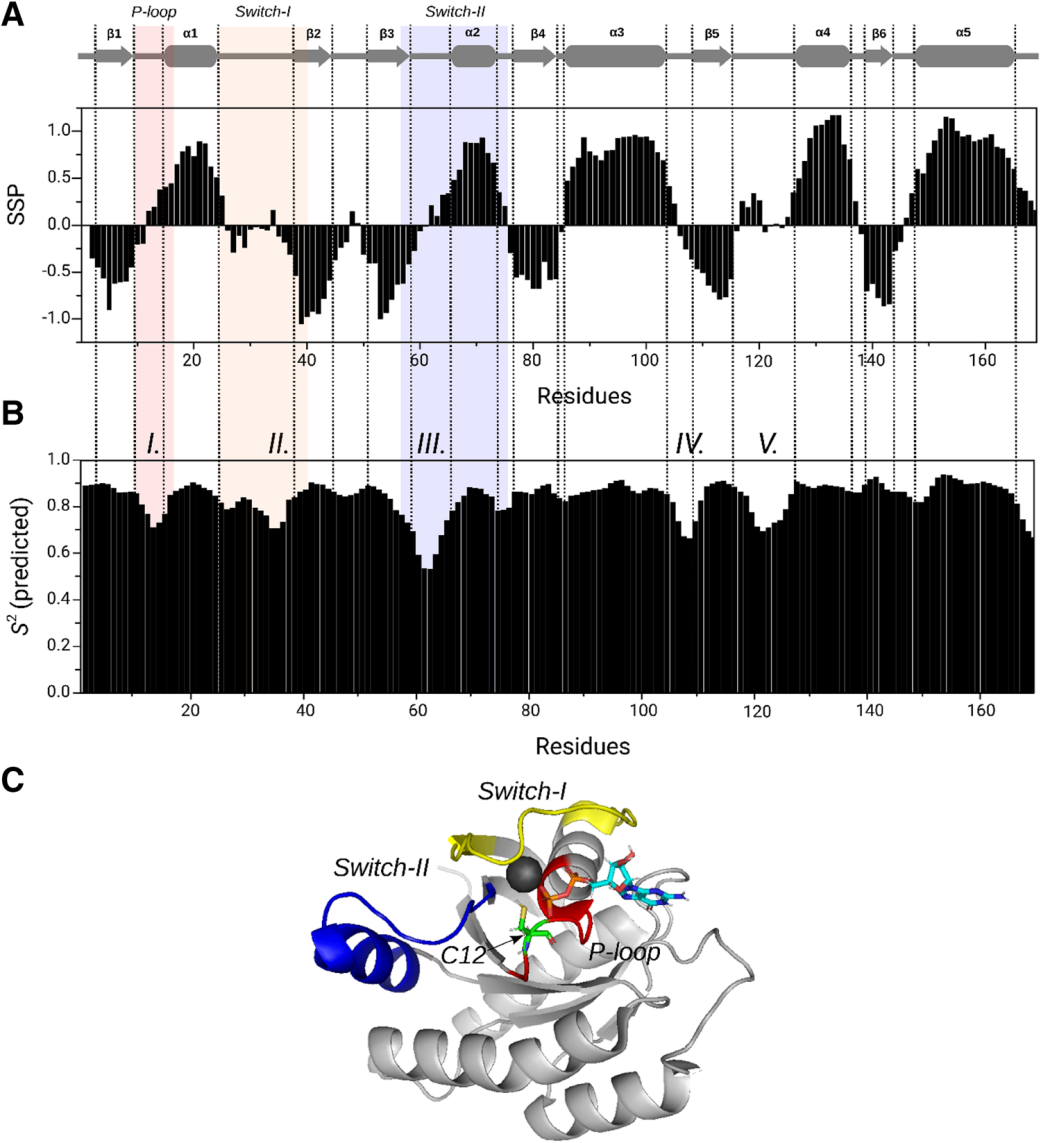
Prediction of secondary structure and backbone dynamics calculated from the assigned chemical shifts of K-Ras4B-G12C-GDP: **a** secondary structure by SSP values and **b** generalized order parameters (*S*^2^) derived from random coil index along the sequence. All the six β-sheets (SPP < − 0.4, β1–β6 indicated above the graphs) and five α-helices (SPP > 0.4, α1–α5 also above the panel) are clearly distinguishable on the SSP-graph (**a**), while the five flexible regions form (indicated as I–V. above the panel) on the *S*^2^ graph (**b**). P-loop (G10–S17 residues) is red-colored, Switch-I (Q25–Y40 residues) is orange-colored and Switch-II (D57–G75 residues) is blue-colored. **c** 3D cartoon structure of K-Ras4B-G12C-GDP (PDB code: 4LDJ), color codes: P-loop is red, Switch-I is yellow, Switch-II is blue, Mg^2+^ ion is dark grey, GDP is cyan and the mutated site, C12 is green (located in P-loop)

Using the G12C mutant ^13^C^α^, ^13^C^β^ and ^1^H^α^ resonances the secondary structure of the protein was predicted via its SSP values (Marsh et al. 2006). The well-known secondary structure information are as follows (Fig. 2a), by taking the definition of α-helices as SPP > 0.4 and β-sheets as SSP < − 0.4: β1 sheet E3–V9, α1 helix G15–I24, β2 sheet D38–V44, β3 sheet C51–T58, α2 helix A66–R73, β4 sheet G77–I84, α3 helix N86–K104, β5 sheet V109–G115, α4 helix T127–S136, β6 sheet I139–E143 and α5 helix T148–K165. These secondary structures with their boundaries agree with those derived from the crystal structure (PDB code: 4LDJ). Only for a few residues we have found some minor differences in terms of boundary shift (e.g. α1 helix: G15–N26, β2 sheet D38–I46, α2 helix: Y64–G75, β4 sheet: G77–A83 in 4LDJ structure). Utilizing the measured chemical shifts of ^13^C^α^, ^13^C^β^, ^13^C’, ^1^H^α^, ^1^H^N^, ^15^N^H^ nuclei backbone dynamics can be characterized for the G12C mutant based on the random coil index (RCI) introduced by Berjanskii and Wishart (Berjanskii and Wishart 2005). The predicted generalized order parameters (*S*^2^ values) calculated from RCI values (Fig. 2b**)** reveal that five flexible regions can be distinguished, namely C12–G15 (P-loop), D33–I36 (Switch-I), D57–S65 (Switch-II), S106–V109 and D119–T124 (*S*^2^ value of 0.78 was used as a limit). The latter two regions are not involved in GDP binding directly, thus they can be allosteric regions if they affect the function of the K-Ras4B-G12C. (P-loop, Switch-I and Switch-II regions are shown in Fig. 2c on the 3D structure of the G12C mutant.)

## The comprehensive analysis of the wt and G12C, G12D, G12V mutants

**Fig. 3:**
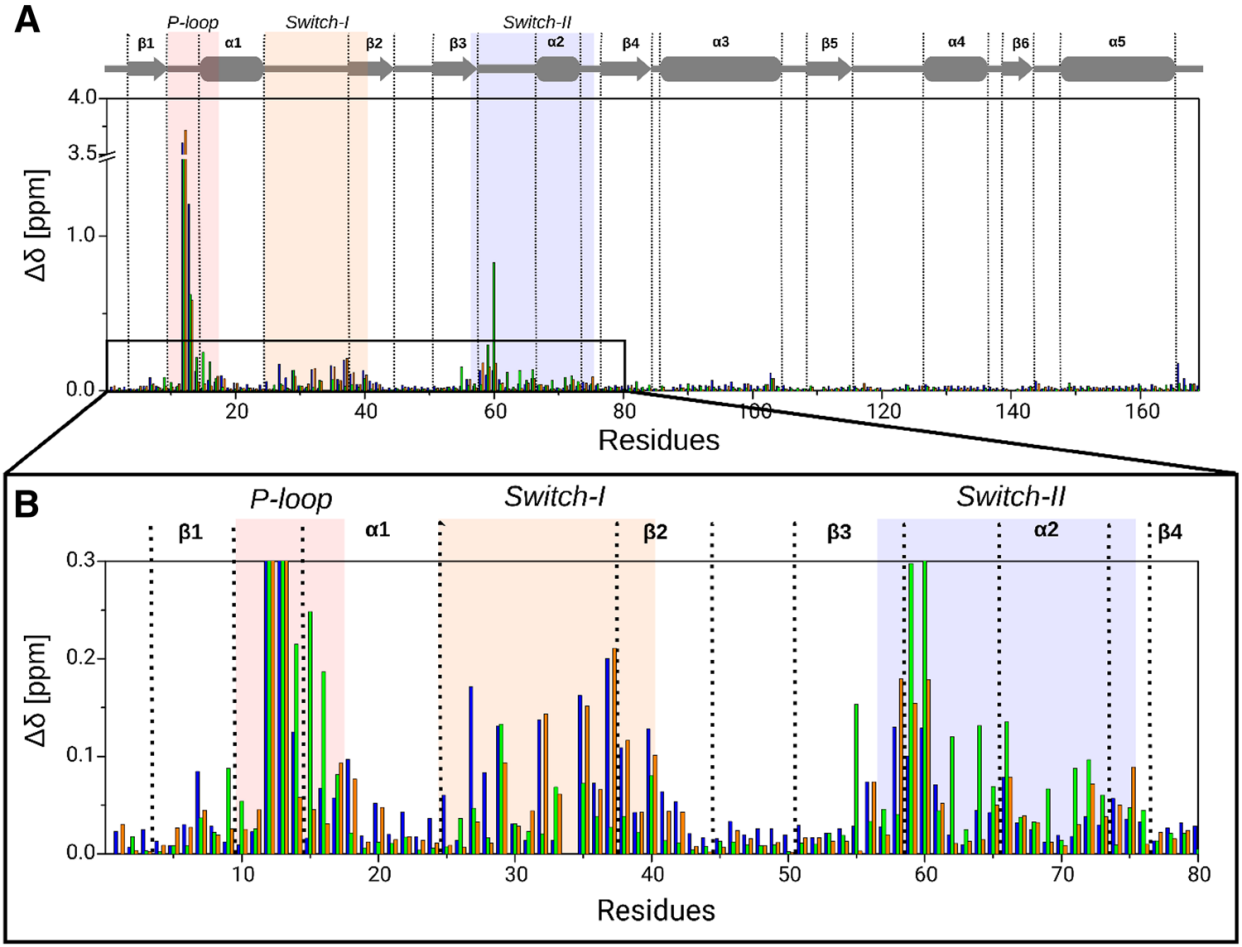
The combined chemical shift differences, Δ*δ*, between the K-Ras4B-wt and the mutants show the structural difference introduced by G12 mutation: blue color stands for G12C, green for G12D and orange for G12V. **a** The P-loop, Switch-I and Switch-II regions differ more extensively, while the rest of the protein is rather similar. **b** Residues M1–C80 highlighted to show that G12C and G12V are more similar (blue and orange) to each other while G12D is more different from them especially concerning the P-loop (G10–S17 residues) and Switch-II (D57–G75 residues). G12D has low Δ*δ* values in Switch-I (Q25–Y40 residues) showing that in this region G12D has the most similar structure to the wt among the three mutants

The effect of G12 mutation on the overall structure can be quantified based on the ^1^H and ^15^N chemical shifts. The combined chemical shift differences, *Δδ* were calculated for all of the residues as follows:

1 $$\Delta \delta = \sqrt {0.5\left[ {\left( {\Delta \delta_{\text{H}} } \right)^{2} + 0.14\left( {\Delta \delta_{\text{N}} } \right)^{2} } \right]}$$

where *Δδ*_H_ and *Δδ*_N_ are the differences in terms of ^1^H and ^15^N chemical shifts, respectively, between a mutant and the wild type protein. (The scaling factor 0.14 used in Eq. 1 was recommended by Williamson 2013.) We have detected only a slight variation in the allosteric lobe (Fig. 3a), while more significant differences were found in P-loop, Switch-I and Switch-II regions of the effector lobe (Fig. 3b). As expected, the largest difference was found for position 12 as well as for the neighboring G13. Interestingly, resonances of G12C and G12V mutants are highly similar to each other and tend to differ from those of the wt protein in P-loop, Switch-I and Switch-II regions. The above similarities are quite visible when overlaying the appropriate ^1^H, ^15^N-HSQC spectra (Fig. 1): indeed only minor differences could be found between resonances of G12C and G12V mutants. In contrast to the above, resonances of G12D mutant are very different from those of either the wt or the other two mutants in P-loop and Switch-II regions, where *Δδ* values of G12D mutant are mostly increased. However, in Switch-I G12D mutant has only slightly different values from the wt and thus differs more from G12C and G12V mutants **(**Fig. 3b**)**. The differences in chemical shifts of ^1^H and ^15^N tend to rise from mainly electronic or structural variations among other effects. In the P-loop probably electronic effect is the main reason due to a residue change affecting the neighboring residues too. Nevertheless, Switch-I and Switch-II regions are more distant from the mutation site in the sequence, therefore mainly structural changes can cause the deviation in chemical shifts. Our results suggest that the structure of G12C and G12V mutants are very similar to each other in the nucleotide binding pocket in GDP-bound form involving Switch-I and Switch-II regions but they differ from the wt and G12D mutant. Meanwhile, G12D mutant structure is very similar to the wt in Switch-I region but highly different from that in Switch-II region.

To conclude we found as follows:

1. Only minor changes occur in terms of chemical shift and thus secondary structure in the catalytic domain of K-Ras4B due to the mutation of G12.
2. Both G12C and G12V mutants are very similar to each other, differing from the wildtype in the P-loop, Switch-I and Switch-II only.
3. The G12D mutant deviates from the wildtype more in the P-loop and Switch-II, and they are more similar to each other in the Switch-I region.
4. The difference in chemical shifts derives from the change of one residue, G12 in the P-loop (electronic effects) while it rises from structural difference in Switch-I and Switch-II meaning that in the two switch regions G12C and G12V mutants are structurally similar to each other and differ from wildtype and G12D mutant. G12D mutation does not affect structure of the Switch-I region significantly but causes changes in that of Switch-II.
5. Former studies (Hunter et al. 2015) concluded that the GTP autohydrolysis rate of G12C is similar to that of the wildtype. Furthermore, that of G12V mutant turned out to be the smallest, while that of G12D mutant is between them. Comparing these results with our findings, the order of the hydrolysis rates strongly disagree with the structural similarity of the hydrolysis resulting GDP-bound form. Although in the hydrolysis GTP-bound forms play the main role, but it also depends on the resulting GDP-bound forms (aim of the present study) as well. Thus our investigation reveals that the mechanism of the autohydrolysis must be related not only to the overall structure, but to additional aspects of the proteins, such as internal motion (backbone dynamics). Structure related backbone dynamics seems to be a plausible presumption, as it is known that dynamics could indeed play an important role in „molecular switches” (Mott and Owen 2018).
